# COVID-19 vaccination uptake amongst ethnic minority communities in England: a linked study exploring the drivers of differential vaccination rates

**DOI:** 10.1093/pubmed/fdab400

**Published:** 2022-01-06

**Authors:** Charlotte Hannah Gaughan, Cameron Razieh, Kamlesh Khunti, Amitava Banerjee, Yogini V Chudasama, Melanie J Davies, Ted Dolby, Clare L Gillies, Claire Lawson, Evgeny M Mirkes, Jasper Morgan, Karen Tingay, Francesco Zaccardi, Thomas Yates, Vahe Nafilyan

**Affiliations:** Office for National Statistics, Newport NP10 8XG, UK; Diabetes Research Centre, University of Leicester, Leicester General Hospital, Leicester LE5 4PW, UK; National Institute for Health Research (NIHR) Leicester Biomedical Research Centre (BRC), Leicester General Hospital, Leicester LE5 4PW, UK; Leicester Real World Evidence Unit, Diabetes Research Centre, University of Leicester, Leicester LE5 4PW, UK; Diabetes Research Centre, University of Leicester, Leicester General Hospital, Leicester LE5 4PW, UK; Leicester Real World Evidence Unit, Diabetes Research Centre, University of Leicester, Leicester LE5 4PW, UK; NIHR Applied Research Collaboration – East Midlands (ARC-EM), Leicester General Hospital, Leicester LE5 4PW, UK; Leicester Diabetes Centre, University Hospitals of Leicester NHS Trust, Leicester General Hospital, Leicester LE5 4PW, UK; Institute of Health Informatics, University College London, London NW1 2DA, UK; Department of Cardiology, University College London Hospitals NHS Foundation Trust, London NW1 2PG, UK; Department of Cardiology, Barts Health NHS Trust, London E1 1BB, UK; Diabetes Research Centre, University of Leicester, Leicester General Hospital, Leicester LE5 4PW, UK; Leicester Real World Evidence Unit, Diabetes Research Centre, University of Leicester, Leicester LE5 4PW, UK; NIHR Applied Research Collaboration – East Midlands (ARC-EM), Leicester General Hospital, Leicester LE5 4PW, UK; Leicester Diabetes Centre, University Hospitals of Leicester NHS Trust, Leicester General Hospital, Leicester LE5 4PW, UK; Diabetes Research Centre, University of Leicester, Leicester General Hospital, Leicester LE5 4PW, UK; National Institute for Health Research (NIHR) Leicester Biomedical Research Centre (BRC), Leicester General Hospital, Leicester LE5 4PW, UK; Leicester Diabetes Centre, University Hospitals of Leicester NHS Trust, Leicester General Hospital, Leicester LE5 4PW, UK; Office for National Statistics, Newport NP10 8XG, UK; Diabetes Research Centre, University of Leicester, Leicester General Hospital, Leicester LE5 4PW, UK; Leicester Real World Evidence Unit, Diabetes Research Centre, University of Leicester, Leicester LE5 4PW, UK; NIHR Applied Research Collaboration – East Midlands (ARC-EM), Leicester General Hospital, Leicester LE5 4PW, UK; Leicester Diabetes Centre, University Hospitals of Leicester NHS Trust, Leicester General Hospital, Leicester LE5 4PW, UK; Leicester Real World Evidence Unit, Diabetes Research Centre, University of Leicester, Leicester LE5 4PW, UK; Department of Cardiovascular Sciences, University of Leicester, Leicester LE3 9QP, UK; School of Computing and Mathematical Science, University of Leicester, Leicester LE1 7RH, UK; Office for National Statistics, Newport NP10 8XG, UK; Office for National Statistics, Newport NP10 8XG, UK; Diabetes Research Centre, University of Leicester, Leicester General Hospital, Leicester LE5 4PW, UK; Leicester Real World Evidence Unit, Diabetes Research Centre, University of Leicester, Leicester LE5 4PW, UK; Diabetes Research Centre, University of Leicester, Leicester General Hospital, Leicester LE5 4PW, UK; National Institute for Health Research (NIHR) Leicester Biomedical Research Centre (BRC), Leicester General Hospital, Leicester LE5 4PW, UK; Office for National Statistics, Newport NP10 8XG, UK; Faculty of Public Health, Environment and Society, London School of Hygiene and Tropical Medicine, London WC1E 7HT, UK

**Keywords:** COVID-19, cultural, demographic, ethnicity, social, sociodemographic factors, vaccination

## Abstract

**Background:**

Despite generally high coronavirus disease 2019 (COVID-19) vaccination rates in the UK, vaccination hesitancy and lower take-up rates have been reported in certain ethnic minority communities.

**Methods:**

We used vaccination data from the National Immunisation Management System (NIMS) linked to the 2011 Census and individual health records for subjects aged ≥40 years (n = 24 094 186). We estimated age-standardized vaccination rates, stratified by ethnic group and key sociodemographic characteristics, such as religious affiliation, deprivation, educational attainment, geography, living conditions, country of birth, language skills and health status. To understand the association of ethnicity with lower vaccination rates, we conducted a logistic regression model adjusting for differences in geographic, sociodemographic and health characteristics. **Results**

All ethnic groups had lower age-standardized rates of vaccination compared with the white British population, whose vaccination rate of at least one dose was 94% (95% CI: 94%–94%). Black communities had the lowest rates, with 75% (74–75%) of black African and 66% (66–67%) of black Caribbean individuals having received at least one dose. The drivers of these lower rates were partly explained by accounting for sociodemographic differences. However, modelled estimates showed significant differences remained for all minority ethnic groups, compared with white British individuals.

**Conclusions:**

Lower COVID-19 vaccination rates are consistently observed amongst all ethnic minorities.

## Introduction

Vaccination programmes are currently taking place to help address the ongoing coronavirus disease 2019 (COVID-19) pandemic. Vaccination is currently the principal strategy to mitigate against the effects of COVID-19 at population level, beyond social distancing measures. In the UK vaccination rates are amongst the highest in the world, with take-up rates estimated to be around 90%.^1^^,^^2^ However, within the UK, implementation of the COVID-19 vaccination programme has demonstrated disparities in vaccination uptake between certain population groups. Preliminary reports suggest ethnicity has been found to be an important factor in vaccination uptake, with minority ethnic groups reporting lower uptake.^3^^,^^4^ This is particularly concerning as minority ethnic groups have consistently been found to experience higher levels of morbidity and mortality due to the COVID-19 pandemic in the UK.^5^^,^^6^

Current understanding of the reasons why vaccination uptake is lower in minority ethnic groups is limited and poorly understood. Several studies have reported that, in the UK, intention to be vaccinated for COVID-19 is lower in minority ethnic groups compared with white individuals.^7^^,^^8^ In addition, historical vaccination programmes have demonstrated poorer uptake in minority ethnic communities.^9^^,^^10^ In some previous studies investigating vaccination uptake more broadly, sociodemographic and cultural factors such as deprivation, religious affiliation and household composition may explain the lower uptake in some minority ethnic groups.^3^^,^^11^^,^^12^ However, few studies to date have been able to investigate real-time COVID-19 vaccination rates between ethnic groups and the relationship between uptake and social and cultural factors. Furthermore, existing studies have been unable to explain why uptake may be lower in certain minority ethnic groups, which requires further investigation. Given that vaccination uptake in ethnic groups is likely determined by many potentially interacting historical, sociodemographic and cultural factors, it is important to conduct a detailed investigation at population level.

Therefore, the aim of this study was to assess the uptake of COVID-19 vaccination in people ≥40 years of age in England using population-level vaccination records linked to the 2011 Census and clinical records. We provide an in-depth analysis of how uptake varies between ethnic groups and investigated the impact of sociodemographic and cultural factors which may act as important explanatory factors in this relationship, such as religious affiliation, deprivation, educational attainment, geography, living conditions, country of birth, language skills and presence of underlying health conditions.

**Table 1:** Distributions of the study population

Variable	Level	All people %	Number
Age (years)	Mean	58.3	14 046 910
Sex	Male	47.2	11 372 456
	Female	52.8	12 721 730
Ethnicity	White British	84.0	20 239 116
	White Other	5.3	1276 992
	Bangladeshi	0.5	120 471
	Pakistani	1.5	361 413
	Black African	1.3	313 224
	Black Caribbean	1.1	265 036
	Chinese	0.5	120 471
	Indian	2.5	602 355
	Mixed	1.0	240 942
	Other	2.3	554 166
Religion	Christian	64.9	15 637 127
	Buddhist	0.5	120 471
	Hindu	1.5	361 413
	Jewish	0.5	120 471
	Muslim	3.4	819 202
	Sikh	0.7	168 659
	Other religion	0.5	120 471
	Religion not Stated	6.5	1566 122
	No Religion	21.6	5204 344
Urban–Rural	Rural	20.4	4915 214
	Urban	79.6	19 178 972
Region	North East	5.0	1204 709
	North West	13.3	3204 527
	Yorkshire and the Humber	9.9	2385 324
	East Midlands	8.9	2144 383
	East England	10.6	2553 984
	London	11.6	2794 926
	South East	13.3	3204 527
	South West	16.8	4047 823
	North West	10.7	2578 078
Tenure of household	Owned outright	74.3	17 901 980
	Social rented	12.8	3084 056
	Private rented	11.0	2650 360
	Other	1.9	457 790
Level of highest qualification	No qualification	21.5	5180 250
	Level 1: 1–4 GCSE/O-Level	13.8	3324 998
	Level 2: 5+ GCSE/O levels	14.1	3397 280
	Level 3: 2+ A Levels or equivalent	10.3	2481 701
	Level 4+: degree or above	30.7	7396 915
	Other	5.8	1397 463
Index of Multiple Deprivation (IMD)	Quintile 1 (most deprived)	17.0	4096 012
	Quintile 2	19.2	4626 084
	Quintile 3	20.7	4987 497
	Quintile 4	21.4	5156 156
	Quintile 5 (least deprived)	21.9	5276 627
Long-term health problem or disability	Daily activities not limited	81.2	19 564 479
	Daily activities limited a little	11.3	2722 643
	Daily activities limited a lot	7.4	1782 970

## Methods

### Data and Study Population

To estimate COVID-19 vaccination take-up amongst ethnic minority groups in England, we linked vaccination data from the National Immunisation Management System (NIMS) to the Office for National Statistics (ONS) Public Health Data Asset (PHDA). NIMS contains vaccination records for >60 million people across the UK. The PHDA combines the 2011 Census, General Practice Extraction Service (GPES) data and Hospital Episode Statistics (HES). The linkage was conducted by using individuals’ unique National Health Service (NHS) number. The Census was linked to the 2011–2013 NHS Patient Registers using deterministic and probabilistic matching, with a linkage rate of 94.6%.

The study population consisted of people aged 40 years and over, alive on 15 June 2021 who were resident in England, registered with a general practitioner, and were enumerated at the 2011 Census. Out of the 26 204 991 adults aged 40 or over who received a first dose of a COVID-19 vaccine in NIMS, 84.5% were linked to the ONS PHDA. By 15 June 2021, those 40 and over should have been offered at least one dose of a COVID vaccine.

### Outcome Variable

Our outcome of interest is having received at least one COVID-19 vaccine by 15 June 2021, as recorded in the NIMS data.

### Exposure variable

Using voluntary self-reported ethnic affiliation from the 2011 Census, individuals were classified into one of the 10 groups: white British, Bangladeshi, black African, black Caribbean, Chinese, Indian, mixed, other, Pakistani, white other.

### Covariates

All individual-level sociodemographic characteristics (age, sex, household tenure, religious affiliation, disability status, educational attainment, country of birth, main language) were taken from the 2011 Census. Place of residence (region, urban rural) and area-based deprivation (Index of Multiple Deprivation) were derived based on data from the 2019 Patient Register. Health conditions were defined as comorbidities identified in the QCovid risk prediction model.^13^ Health conditions included body mass index, diabetes, coronary heart disease, stroke and congestive cardiac failure, which are known to differ by ethnicity.^14–17^ A full list of health conditions included can be found in the legend of Fig. 2.

### Statistical analyses

We calculated age-standardized vaccination rates for ethnic groups whereby the age-by-sex distribution within each ethnic group was standardized to the overall distribution in the study population. This enabled us to compare vaccination rates between ethnic groups, accounting for differences in age and sex structure. These rates were further stratified by the covariates listed above excluding health, enabling us to identify specific demographic groups where vaccination rates were lower.

To understand the drivers of the observed differences in vaccination rates between ethnic groups, we conducted a logistic regression. We estimated the odds of not receiving one dose of a COVID-19 vaccine in minority ethnic groups compared with the white British group. We accounted for different characteristics by incrementally adding (i) age (fitted as a cubic spline) and sex (ii) geographic location including rural–urban location to account for possible access barriers, (iii) a range of socioeconomic status variables (household tenure, educational attainment, index of multiple deprivation) as these have been shown to be associated with lower vaccination uptake; and (iv) disability status and health conditions owing to individual concerns that the vaccination may adversely affect underlying health conditions and possible concerns surrounding accessibility.

We tested for interactions by comparing model fits and interaction plots and present significant interactions as separate stratified models (country of birth and main language). Descriptions of the models can be found in the supplementary material. We further examined the ethnic breakdown of the UK population from 2012 to 2019 to assess whether the ethnic breakdown of the population is stable over time.

## Results

The study population consisted of 24 094 186 adults (52.9% women) living in England, mean age 58.3 years. There were 20 315 646 (84.3%) white British, 1200 376 (5%) white other, 617 994 (2.6%) Indian, 362 632 (1.5%) Pakistani, 129 298 (0.5%) Bangladeshi, 314 469 (1.3%) black African, 262 792 (1.1%) black Caribbean, 120 426 (0.5%) Chinese, 232 794 (1%) mixed and 537 759 (2.2%) other ethnicity individuals. The descriptive details of the cohort are shown in Table 1. Overall, 22 143 218 (92%) people received at least one dose of a vaccine by 15 June 2021, with the vaccination programme in the UK beginning on 8 December 2020.

**Fig. 1:**
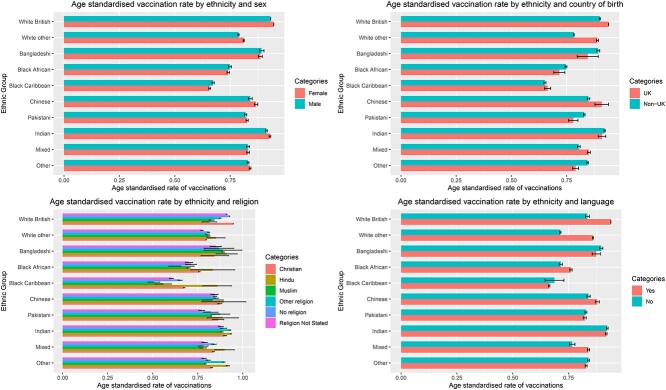

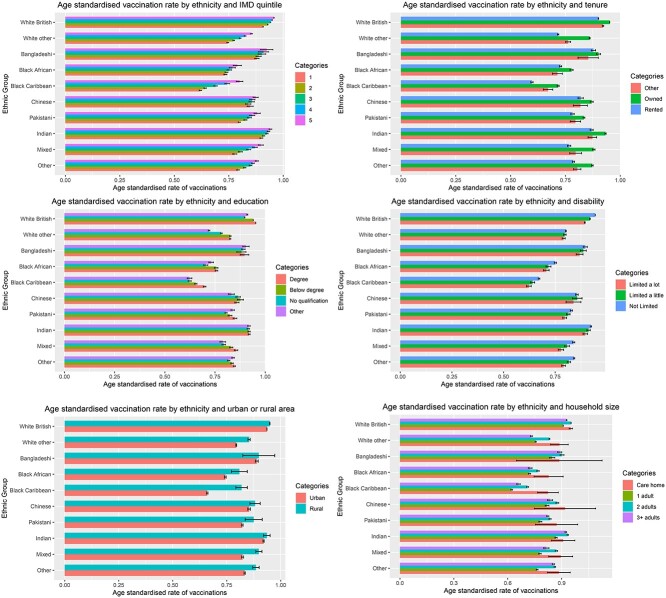
Age-standardized rates. Language yes = main language was English; language no = main language was not English.tenure = household tenure.

Age-standardized vaccination rates varied markedly between ethnic groups across a range of sociodemographic factors (Fig. 1). All minority ethnic groups had lower vaccination uptake compared with white British individuals, with uptake consistently being lowest amongst black African and black Caribbean individuals across the range of sociodemographic factors. Age-standardized vaccination rates not stratified across any sociodemographic factors can be found in Table S1.

While vaccination rates were lower in minority ethnic groups, patterns of vaccination across most sociodemographic factors were generally similar between ethnic groups, with greater area deprivation, lower educational attainment, less advantaged socioeconomic position, living in urban environments, living alone and being severely limited by a disability, all showing lower vaccination uptake across all ethnic groups. However, some of these differences were more pronounced in black African and black Caribbean ethnic groups compared with other ethnic groups. For example, in the most deprived black African and black Caribbean individuals, age-standardized vaccination rates were 73.4% (72.7%, 74.1%) and 62% (61.4%, 62.5%), whereas the most deprived Bangladeshi, Chinese, Indian, Pakistani, white British and white other ethnicities were 87.9% (86.9%, 88.9%), 84.8% (83.4%, 86.2%), 90% (89.3%, 90.6%), 79.9% (79.4%, 80.4%), 90.8% (90.7%, 90.9%) and 74.5% (74.1%, 74.9%) (Fig. 1), respectively. Black African and black Caribbean individuals living in an urban environment had a vaccination uptake of 74.4% (73.9%, 74.8%) and 66% (65.7%, 66.4%) respectively, while their black African and black Caribbean counterparts living in rural areas had an uptake of 80.9% (77.2%, 84.6%) and 81.9% (79.2%, 84.6%), respectively. In comparison, for white British, Indian, Pakistani and Bangladeshi individuals living in urban environments, uptake was 93.7% (93.6%, 93.7%), 92.1% (91.8%, 92.4%), 82.2% (81.9%, 82.6%) and 88.9% (88.3%, 89.6%), while uptake for their counterparts living in rural environments was 95% (94.9%, 95.1%), 93.6% (92.1%, 95.1%), 87.5% (83.6%, 91.5%) and 89.9% (82.5%, 97.3%) (Fig. 1).

South Asian, black African and other ethnicities born in the UK had lower vaccination rates than their counterparts born abroad. In black African, Chinese, mixed, white British and white other groups, vaccination was higher in those whose main language was English compared with those whose main language was not English. In Bangladeshi, black Caribbean, Indian, other ethnicity and Pakistani people, vaccination rates were higher in those whose main language was not English.

For religious affiliation, vaccination uptake differed between ethnic groups. Lowest uptake was found in black Caribbean individuals who identified as Muslim or ‘other’ religion, with highest uptake found in white British individuals who identified as Christian. In most ethnic groups, Muslim individuals tended to have lower uptake (compared with Christian, Hindu and no religion). There were no clear differences in vaccination for sex between ethnic groups (Table S1).

**Fig. 2:**
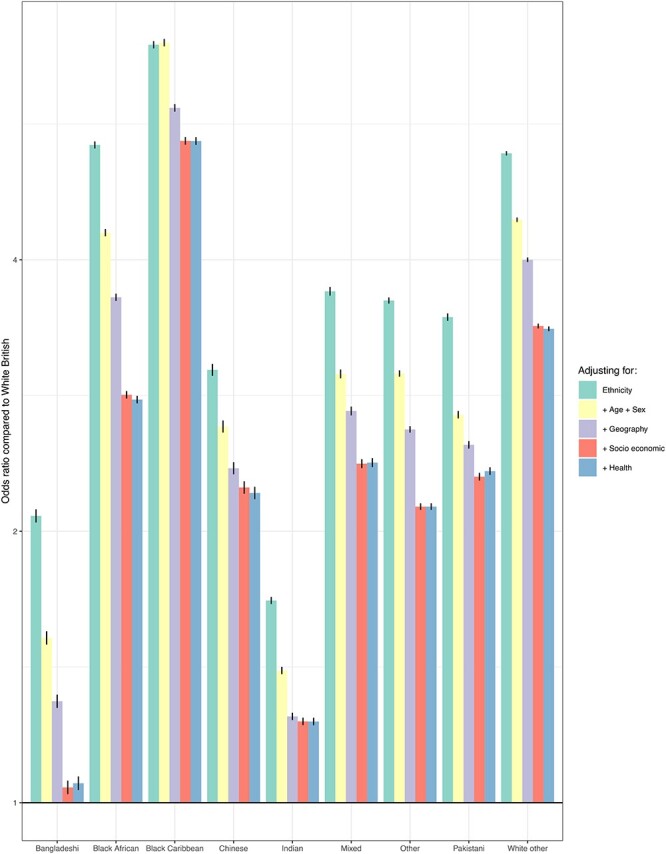
Mediators of the relationship between ethnicity and vaccination rate. Model 1: ethnicity. Model 2: model 1 + age and sex. Model 3: model 2 + region and urban/rural classification. Model 4: model 3 + index of multiple deprivation, household tenure, educational attainment. Model 5: model 4 + disability status + Body mass index (kg/m^2^), chronic kidney disease (no chronic kidney disease, stage 3, stage 4 or stage 5—patients with stage 5 chronic kidney disease were assigned the coefficient for stage 5 disease without transplant nor dialysis), learning disability (no learning disability, Down Syndrome or other learning disability), chemotherapy in the past 12 months (chemotherapy group A, B or C, based on the risk of grade 3 or 4 febrile neutropenia [Common Terminology Criteria for Adverse Events version 4] or lymphopenia), respiratory cancer, radiotherapy in the past 6 months, solid organ transplant, prescribed immunosuppressant medication by general practitioner, prescribed leukotriene or long-acting β2 agonists, prescribed regular prednisolone, diabetes (for the validation of the QCovid risk model, all patients with diabetes were assigned the coefficient type 2 diabetes), chronic obstructive pulmonary disease, asthma, rare pulmonary diseases, pulmonary hypertension or pulmonary fibrosis, coronary heart disease, stroke, atrial fibrillation, congestive cardiac failure, venous thromboembolism, peripheral vascular disease, congenital heart disease, dementia, Parkinson’s disease, epilepsy, rare neurological conditions, cerebral palsy, severe mental illness (bipolar disorder, schizophrenia, or severe depression), osteoporotic fracture, rheumatoid arthritis or systemic lupus erythematosus and cirrhosis of the liver.

Figure 2 and Table S2 show odds ratios (ORs) for vaccination by ethnic group from different models. All minority ethnic groups had a lower probability of receiving a vaccine than white British individuals in unadjusted models, with the probability of not receiving a vaccine being particularly pronounced in black African (OR: 5.36; 95% CI: 5.32-5.40) and black Caribbean (6.93; 6.87-6.98) individuals. Adjusting for differences in age, sex, geography, deprivation and education explained some but not all of the differences in uptake between people from minority ethnic groups and white British individuals (e.g. black African: 2.80 (2.78-2.82); black Caribbean: 5.42 (5.37–5.46)). Further adjusting for underlying health status did not meaningfully change the results compared with adjusting for differences in deprivation and education in any minority ethnic group. Results stratified by cultural factors, such as country of birth and English language skills, can be found in Tables S3 and S4. The ethnic breakdown of the UK population was found to be relatively stable from 2012 to 2019 (Table S5).

## Discussion

In over 24 million adults aged over 40 years in England with population-level linked data, we found that the uptake of a first dose of COVID-19 vaccine was lower amongst all minority ethnic groups compared with the white British ethnic group, with differences being particularly pronounced in black African and black Caribbean individuals. Differences in the likelihood of receiving a first dose vaccine remained after adjusting for a wide range of sociodemographic factors, including age, sex, geography, deprivation, educational attainment and underlying health condition across all ethnic groups. Similar patterns were found when examining age-standardized vaccination rates across a wide range of sociodemographic factors, with vaccination rates being lower in all minority ethnic groups. However, some stark disparities within some sociodemographic factors were found. The difference in vaccination uptake between the most and least deprived black Caribbean individuals was far greater (18%) compared with other ethnic groups (e.g. 5% for white British). In addition, black African and black Caribbean individuals who lived in urban environments had markedly lower uptake than all other ethnic groups.

To our knowledge, this is the largest population-level study to specifically investigate the drivers of ethnic disparities in COVID-19 vaccination in England across a wide range of sociodemographic factors. Our findings are consistent with previous evidence in an initial study examining the trends and clinical characteristics of people receiving a COVID-19 vaccine,^2^ a population-based study investigating sociodemographic inequality in COVID-19 vaccination amongst elderly adults in England^3^ and with evidence examining historical vaccination programmes,^9^^,^^10^ which all found that the proportion of ethnic minorities vaccinated was lower than white British individuals. We extend these previous studies by investigating the drivers of these ethnic disparities. We examined vaccination rates across a wide range of sociodemographic factors stratified by ethnicity. Our findings suggest that black African and particularly black Caribbean individuals who are more deprived and live in urban environments have at high probability of not receiving a first dose of a COVID-19 vaccine. This is supported by previous research that has found that deprivation is independently associated with lower probability to receive various types of vaccines.^18^^,^^19^ Current evidence suggests that while the ethnic disparities in vaccination have narrowed in recent months, inequalities between certain ethnic groups still persist.^4^^,^^20^ However, this study suggests that investigating how ethnicity interplays with key sociodemographic factors, such as deprivation, geography, religion and language skills may provide greater identification of subgroups at high-risk of not receiving a vaccine and understanding why certain subsets of ethnic groups may have lower vaccination uptake.

We found that accounting for differences in age, sex, geography, deprivation, educational attainment and underlying health status reduces the differences in vaccine uptake between people from all minority ethnic groups compared with white British individuals. Importantly, however, adjusting for these factors did not completely explain the differences in vaccination status, with the adjusted odds of not receiving a vaccine still higher in all minority ethnic groups.

When examining country of birth and language skills, variation was found between ethnic groups for COVID-19 vaccination uptake. This suggests that in some minority ethnic groups, cultural factors are likely to be important drivers of vaccine disparities. However, further research is required to clarify this. In addition, we found that people who identified as Muslim (compared with Christian, Hindu or no religion) had lower vaccination uptake across most ethnic groups, which supports previous evidence suggesting that factors such as religion may be important in determining vaccination uptake.^11^ This suggests that engaging with certain religious groups and their leaders about vaccination within community/religious settings may potentially improve issues around potential initial vaccine hesitancy, logistics of getting vaccinated and any language/cultural barriers. This may further suggest that the social capital of religion may be useful in increasing vaccination uptake.^21^

Importantly, it has previously been reported that minority ethnic groups have higher risk of developing severe COVID-19, COVID-19 complications and related mortality,^5^^,^^6^^,^^22^ with findings stating that some of the elevated risk is explained by some of the same sociodemographic variables that we include in this study. Therefore, the individuals who are at most risk are least likely to take up vaccination (despite prioritization in the vaccination programme). This reinforces that increasing vaccination amongst minority ethnic groups and subsequent subsets of minority ethnic groups is a public health priority.

The primary strength of this study is using nationwide linked population-level data from clinical records and the 2011 Census. Unlike studies based solely on electronic health records, we examined a wide range of sociodemographic characteristics by ethnicity, which are likely to be important in understanding vaccination trends in these groups. In contrast to the majority of existing data sources, we can precisely estimate vaccination rates and odds ratios for small groups. In addition, we have large numbers of people from ethnic minority backgrounds and were therefore able to stratify by small ethnic minority groups, which is more representative of contemporary Britain. Further, as ethnicity coding is derived from the Census and therefore self-reported, it should be a more reliable method to measure ethnicity than data collected in electronic healthcare records. Most other studies to date do not have this level of detail on ethnicity.

However, this study does have some important limitations, the main being that most demographic and socioeconomic characteristics are derived from the 2011 Census and are 10 years old. However, we focused primarily on characteristics that are unlikely to change over time, i.e. ethnicity, religion, educational attainment and country of birth. Furthermore, other characteristics are likely to be stable for our population (adults aged 40), such as household tenure and sociodemographic characteristics. Table S5 shows that the sociodemographic ethnic breakdown of the UK population from 2012 to 2019 is relatively stable over time. Residency and area deprivation were derived from the 2019 Patient register and therefore should be relatively accurate. However, for the characteristics likely to change over time, such as disability status and language skills, the time difference may introduce some bias into the estimates. Further, we do not control for prior infection in communities; evidence suggests ethnic communities in the UK had higher cumulative infection rates than the white British population, therefore prior infection with COVID-19 may be a factor in deciding whether to get vaccinated.^5^ Another limitation related to this is that adults aged 40 years or younger were not included in our study owing to data linking limitations. However, the quality and stability of Census variables in those individuals is likely to be worse, as some of the variables collected are more likely to change in a younger population. As the Public Health Data Asset was based on the 2011 Census, it excluded people living in England in 2011 but not taking part in the 2011 Census, for example, respondents who could not be linked to the 2011–2013 NHS patients register and recent migrants. Furthermore, we did not include second vaccination dose patterns due to the lower number of younger aged adults who would not have had the opportunity to receive two doses during the study period. However, reports suggest that among adults who received a first dose of vaccine against COVID-19 by 15 March 2021, 96% had received a second dose within 2 months,^23^ therefore the results of this study should still be applicable to second dose vaccination. Lastly, it is important to note that our vaccination rates are based on a derived linked dataset, the true denominator of the population in the UK is unknown, and therefore it is possible that other studies may estimate different vaccination take-up rates.

### Conclusion and implications

This population-level study of UK adults aged 40 or over demonstrates that all minority ethnic groups are more likely to have not received a first dose of COVID-19 vaccine compared with white British individuals, even after adjustment for a range of sociodemographic and health factors, with differences particularly pronounced in black African and black Caribbean individuals. We found disparities in vaccination uptake within a host of sociodemographic factors between all ethnic groups, however black African and black Caribbean individuals who were more deprived and living in urban environments were at particularly high risk of not receiving a vaccine, compared with all other ethnic groups. Religious background may additionally play an important role in vaccination uptake between ethnic groups.

These results emphasize the importance of culturally tailored public health policy messaging and community engagement to increase vaccination uptake within ethnic minority communities. This is especially important for black African and black Caribbean individuals as they have consistently been found to be at increased risk of COVID-19 mortality in the first two waves of the pandemic. Further, if the UK enters a new wave of infection, booster vaccines are required to reinforce immunity or some restrictions are placed upon individuals who are unvaccinated, ethnic minorities may be disproportionately affected if their vaccination uptake is low. Global implications of these results may be in understanding whether the disparities seen in vaccination uptake by certain ethnic and sociodemographic groups in this study are also seen in other countries with similar ethnic groups, and whose vaccination programmes are not as far advanced as the UK. The knowledge from our study may assist policymakers involved in vaccination programmes to build trust and confidence in groups who may otherwise initially be hesitant of COVID-19 vaccination.

**Highlights:** ‘Vaccination rates are lower amongst all ethnic minority groups in the UK compared with the white British population’.

‘Modelled estimates suggest differences in sociodemographic profiles explain some but not all of lower take-up rates in ethnic communities’.

‘Culturally tailored public health measures to improve vaccination rates should be targeted to black communities, certain religious groups and people living in deprived areas’.

## Author contributions

C.R., V.N., T.Y., C.H.G. and K.K. developed the research question. V.N., C.H.G., C.R. and T.Y. developed the statistical analysis plan. C.H.G. undertook the statistical analysis with support from V.N.; both had access to the data. C.R. and C.H.G. drafted the manuscript. All authors contributed to the research design and revised the manuscript for important intellectual content.

## Funding

This work was supported by grants from UKRI (MRC)-DHSC (NIHR) COVID-19 Rapid Response Rolling Call (MR/V020536/1) and HDR-UK (HDRUK2020.138). CR, TY and KK are supported by the National Institute for Health Research (NIHR) Applied Research Collaboration East-Midlands (ARC EM) and the NIHR Leicester Biomedical Research Centre (BRC). The views expressed in this publication are those of the authors and not necessarily those of the NHS, the National Institute for Health Research, the Department of Health or Public Health England. AB has received grant support from NIHR, BMA, UKRI and Astra-Zeneca (all unrelated to the current work). The funder/sponsor had no role in the design and conduct of the study; collection, management, analysis and interpretation of the data; preparation, review or approval of the manuscript; and decision to submit the manuscript for publication.

## Conflict of interest

KK is Director for the University of Leicester Centre for Ethnic Health Research, Trustee of the South Asian Health Foundation, national NIHR ARC lead for Ethnicity and Diversity and a member of SAGE and Chair of the SAGE subgroup on ethnicity and COVID-19. Other authors declare no conflicts of interests. AB is a Trustee of the South Asian Health Foundation.

## Ethical approval

Ethical approval was obtained from the National Statistician’s Data Ethics Advisory Committee (NSDEC(20)12).

## Data Availability

Analysed data are controlled by the Office of National Statistics, UK. Data are not yet available, but will be made available on the ONS Secure Research Service for accredited researchers.

## Supplementary Material

Supplementary_tables_v3_fdab400

RECORD_Checklist_vaccination_ethnicity_fdab400

## Notes

**Charlotte Hannah Gaughan**, Statistician

**Cameron Razieh**, Epidemiologist/Statistician

**Kamlesh Khunti**, Professor of Primary Care Diabetes and Vascular Medicine

**Amitava Banerjee**, Professor of Clinical Data Science

**Yogini V Chudasama**, Epidemiologist/Statistician

**Melanie J Davies**, Professor of Diabetes Medicine

**Ted Dolby**, Data Analyst

**Clare L Gillies**, Lecturer in Medical Statistics

**Claire Lawson**, Associate Professor

**Evgeny M Mirkes**, Research Associate

**Jasper Morgan**, Senior Linked Data Manager

**Karen Tingay**, Principal Statistical Methodologist

**Francesco Zaccardi**, Clinical Epidemiologist

**Thomas Yates**, Professor of Physical Activity, Sedentary Behaviour and Health

**Vahe Nafilyan**, Head of Cross-cutting Analysis
