# Supplementary material for: COVID-19 vaccination uptake amongst ethnic minority communities in England: a linked study exploring the drivers of differential vaccination rates
Source: J Public Health (Oxf). 2022 Jan 6;45(1):e65–74. doi: 10.1093/pubmed/fdab400 (PMC8755382; doi:10.1093/pubmed/fdab400)
Supplement: Supplementary_tables_v3_fdab400 [file supplementary_tables_v3_fdab400.docx]

## Supplementary tables

**Supplementary Table 1.** Age standardised rates (and 95% confidence intervals) of having at least one dose of a COVID 19 vaccination between 8 December 2021 and 15 June 2021 in England and Wales.

|  | **Overall** |  | **Females** |  | **Males** |  |
| --- | --- | --- | --- | --- | --- | --- |
| **Bangladeshi** | 0.89 (0.88-0.90) | | 0.89 (0.88-0.90) | | 0.89 (0.88-0.90) | |
| **Black African** | 0.74 (0.74-0.75) | | 0.75 (0.74-0.76) | | 0.74 (0.74-0.75) | |
| **Black Caribbean** | 0.66 (0.66-0.67) | | 0.67 (0.67-0.68) | | 0.66 (0.65-0.66) | |
| **Chinese** | 0.86 (0.85-0.86) | | 0.84 (0.83-0.85) | | 0.87 (0.86-0.87) | |
| **Indian** | 0.92 (0.92-0.92) | | 0.91 (0.91-0.92) | | 0.93 (0.93-0.93) | |
| **Mixed** | 0.83 (0.83-0.84) | | 0.83 (0.82-0.84) | | 0.83 (0.82-0.84) | |
| **Other** | 0.84 (0.83-0.84) | | 0.83 (0.83-0.83) | | 0.84 (0.84-0.84) | |
| **Pakistani** | 0.82 (0.82-0.83) | | 0.82 (0.81-0.82) | | 0.83 (0.82-0.83) | |
| **White British** | 0.94 (0.94-0.94) | | 0.93 (0.93-0.93) | | 0.95 (0.95-0.95) | |
| **White Other** | 0.80 (0.80-0.80) | | 0.79 (0.78-0.79) | | 0.81 (0.81-0.81) | |

**Supplementary Table 2.** Odds ratios (and 95% confidence intervals) for odds of not having at least one dose of a COVID 19 vaccination between 8 December 2021 and 15 June 2021 in England and Wales for each ethnic group compared to White British.

|  | **Model 1** |  | **Model 2** |  | **Model 3** |  | **Model 4** |  | **Model 5** |
| --- | --- | --- | --- | --- | --- | --- | --- | --- | --- |
|  | **Ethnicity** |  | **+Age and Sex** |  | **+Geography Variables** |  | **+Socio economic status variables** |  | **+Health Variables** |
| **Bangladeshi** | 2.08 (2.05-2.11) | | 1.52 (1.50-1.55) | | 1.30 (1.28-1.32) | | 1.04 (1.02-1.06) | | 1.05 (1.03-1.07) |
| **Black African** | 5.36 (5.32-5.40) | | 4.29 (4.25-4.32) | | 3.63 (3.61-3.66) | | 2.83 (2.81-2.86) | | 2.80 (2.78-2.82) |
| **Black Caribb** | 6.93 (6.87-6.98) | | 6.96 (6.91-7.02) | | 5.90 (5.85-5.94) | | 5.42 (5.37-5.46) | | 5.42 (5.37-5.46) |
| **Chinese** | 3.02 (2.98-3.06) | | 2.61 (2.58-2.65) | | 2.35 (2.32-2.38) | | 2.24 (2.20-2.27) | | 2.21 (2.17-2.24) |
| **Indian** | 1.68 (1.66-1.69) | | 1.40 (1.39-1.41) | | 1.25 (1.24-1.26) | | 1.23 (1.22-1.24) | | 1.23 (1.22-1.24) |
| **Mixed** | 3.69 (3.66-3.73) | | 2.99 (2.96-3.02) | | 2.72 (2.69-2.75) | | 2.38 (2.35-2.40) | | 2.38 (2.36-2.41) |
| **Other** | 3.61 (3.58-3.63) | | 2.99 (2.97-3.01) | | 2.59 (2.58-2.61) | | 2.13 (2.12-2.14) | | 2.13 (2.12-2.14) |
| **Pakistani** | 3.46 (3.43-3.48) | | 2.69 (2.67-2.72) | | 2.49 (2.47-2.51) | | 2.30 (2.28-2.32) | | 2.33 (2.31-2.35) |
| **White other** | 5.25 (5.23-5.27) | | 4.43 (4.41-4.45) | | 4.00 (3.98-4.02) | | 3.38 (3.36-3.39) | | 3.35 (3.34-3.37) |

Model 1: ethnicity

Model 2: model 1 + age and sex

Model 3: model 2 + region and urban/rural classification

Model 4: model 3 + index of multiple deprivation, household tenure, educational attainment

Model 5: model 4 + Disability status + Body mass index (kg/m^2^), chronic kidney disease (no chronic kidney disease, stage 3, stage 4, or stage 5 – patients with stage 5 chronic kidney disease were assigned the coefficient for stage 5 disease without transplant nor dialysis), learning disability (no learning disability, Down Syndrome, or other learning disability), chemotherapy in the past 12 months (chemotherapy group A, B, or C, based on the risk of grade 3 or 4 febrile neutropenia [Common Terminology Criteria for Adverse Events version 4] or lymphopenia), respiratory cancer, radiotherapy in the past 6 months, solid organ transplant, prescribed immunosuppressant medication by general practitioner, prescribed leukotriene or long-acting β2 agonists, prescribed regular prednisolone, diabetes (for the validation of the QCovid risk model, all patients with diabetes were assigned the coefficient type 2 diabetes), chronic obstructive pulmonary disease, asthma, rare pulmonary diseases, pulmonary hypertension or pulmonary fibrosis, coronary heart disease, stroke, atrial fibrillation, congestive cardiac failure, venous thromboembolism, peripheral vascular disease, congenital heart disease, dementia, Parkinson's disease, epilepsy, rare neurological conditions, cerebral palsy, severe mental illness (bipolar disorder, schizophrenia, or severe depression), osteoporotic fracture, rheumatoid arthritis or systemic lupus erythematosus and cirrhosis of the liver.

**Supplementary Table 3.** Odds ratios (and 95% confidence intervals) for odds of not having at least one dose of a COVID 19 vaccination between 8 December 2021 and 15 June 2021 in England and Wales for each ethnic group compared to White British, stratified by Country of Birth UK-Not UK.

|  | **UK - Model 1** |  | **UK - Model 2** |  | **Not UK - Model 1** |  | **Not UK - Model 2** |
| --- | --- | --- | --- | --- | --- | --- | --- |
|  | **Ethnicity** |  | **Fully Adjusted** |  | **Ethnicity** |  | **Fully Adjusted** |
| **Bangladeshi** | 2.04 (1.96-2.13) | | 1.57 (1.50-1.63) | | 0.78 (0.76-0.79) | | 0.67 (0.66-0.68) |
| **Black African** | 4.50 (4.40-4.61) | | 3.37 (3.29-3.45) | | 2.26 (2.23-2.29) | | 1.70 (1.68-1.72) |
| **Black Caribb** | 6.89 (6.82-6.97) | | 5.43 (5.37-5.49) | | 3.68 (3.63-3.73) | | 3.06 (3.02-3.11) |
| **Chinese** | 1.34 (1.29-1.40) | | 1.36 (1.30-1.42) | | 1.50 (1.48-1.53) | | 1.35 (1.32-1.37) |
| **Indian** | 1.18 (1.17-1.20) | | 1.20 (1.18-1.21) | | 0.79 (0.78-0.80) | | 0.70 (0.69-0.71) |
| **Mixed** | 2.61 (2.58-2.65) | | 2.17 (2.15-2.20) | | 2.01 (1.98-2.04) | | 1.61 (1.58-1.64) |
| **Other** | 3.61 (3.56-3.67) | | 3.00 (2.96-3.05) | | 1.53 (1.51-1.55) | | 1.19 (1.17-1.20) |
| **Pakistani** | 2.93 (2.88-2.97) | | 2.73 (2.69-2.78) | | 1.39 (1.37-1.41) | | 1.29 (1.27-1.30) |
| **White other** | 2.02 (2.00-2.05) | | 1.81 (1.79-1.84) | | 2.59 (2.57-2.61) | | 2.05 (2.03-2.07) |

**Supplementary Table 4.** Odds ratios (and 95% confidence intervals) for odds of not having at least one dose of a COVID 19 vaccination between 8 December 2021 and 15 June 2021 in England and Wales for each ethnic group compared to White British, stratified by English Language Main Language-Not Main Language.

|  | **Main Language - Model 1** |  | **Main Language - Model 2** |  | **Not Main Language - Model 1** |  | **Not Main Language - Model 2** |
| --- | --- | --- | --- | --- | --- | --- | --- |
|  | **Ethnicity** |  | **Fully Adjusted** |  | **Ethnicity** |  | **Fully Adjusted** |
| **Bangladeshi** | 1.79 (1.74-1.84) | | 1.55 (1.51-1.60) | | 0.51 (0.50-0.53) | | 0.53 (0.51-0.54) |
| **Black African** | 4.04 (4.00-4.08) | | 3.16 (3.13-3.19) | | 1.73 (1.68-1.77) | | 1.54 (1.50-1.58) |
| **Black Caribb** | 6.98 (6.92-7.03) | | 6.04 (5.99-6.09) | | 2.88 (2.66-3.11) | | 2.85 (2.63-3.09) |
| **Chinese** | 2.24 (2.19-2.28) | | 2.31 (2.26-2.36) | | 1.02 (0.99-1.05) | | 1.03 (1.00-1.06) |
| **Indian** | 1.35 (1.33-1.36) | | 1.36 (1.35-1.38) | | 0.50 (0.49-0.51) | | 0.52 (0.51-0.53) |
| **Mixed** | 2.78 (2.75-2.81) | | 2.44 (2.41-2.46) | | 1.58 (1.53-1.63) | | 1.39 (1.35-1.44) |
| **Other** | 3.01 (2.98-3.04) | | 2.73 (2.70-2.76) | | 1.07 (1.04-1.09) | | 0.97 (0.95-0.99) |
| **Pakistani** | 2.72 (2.69-2.75) | | 2.60 (2.57-2.63) | | 0.93 (0.90-0.95) | | 1.00 (0.97-1.02) |
| **White other** | 3.03 (3.01-3.05) | | 2.83 (2.81-2.84) | | 2.27 (2.22-2.32) | | 1.98 (1.93-2.02) |

**Supplementary Table 5.** Socio-economic status in ethnic communities from 2012-2019: Annual Population Survey % of people employed in higher managerial professions.

|  | **% of ethnic population 2012** | **% of ethnic population 2019** |
| --- | --- | --- |
| **White** | 8.3% | 9.7% |
| **Mixed ethnic groups** | 4.8% | 6.3% |
| **Indian** | 13.5% | 14.8% |
| **Pakistani/Bangladeshi** | 4.0% | 5.1% |
| **Chinese** | 11.3% | 16.9% |
| **Black** | 4.6% | 5.7% |
| **Other Ethnic group** | 7.0% | 8.8% |

Higher managerial position was derived from Social Grade of the household reference person variable. Approximate Social Grade is a socioeconomic classification based on the occupation, employment, qualification, and tenure of the household reference person.
